# TRPM4 contributes to cell death in prostate cancer tumor spheroids, and to extravasation and metastasis in a zebrafish xenograft model system

**DOI:** 10.1002/1878-0261.13795

**Published:** 2025-01-16

**Authors:** Florian Bochen, Saurav Subedi, Federico La Manna, Sofia Jarrin, Irida Papapostolou, Marianna Kruithof‐de Julio, Christine Peinelt

**Affiliations:** ^1^ Institute of Biochemistry and Molecular Medicine University of Bern Bern Switzerland; ^2^ Department for BioMedical Research, Urology Research Laboratory University of Bern Bern Switzerland; ^3^ Department of Urology, Inselspital, Bern University Hospital University of Bern Bern Switzerland

**Keywords:** cell death, ion channel, prostate cancer, TRPM4, tumor spheroid, zebrafish

## Abstract

Transient receptor potential melastatin‐4 (*TRPM4*) ion channel expression is upregulated in prostate cancer (PCa), contributing to increased cell proliferation, migration, adhesion, epithelial‐to‐mesenchymal transition, cell cycle shift, and alterations of intracellular Ca^2+^ signaling. GEO2R platform analysis of messenger RNA (mRNA) expression of ~ 6350 genes in normal and malignant prostate tissue samples from 15 PCa patients demonstrates that *TRPM4* expression is upregulated sixfold and is among the most significantly upregulated genes in PCa. We find that absence of TRPM4 reduced PCa tumor spheroid size and decreased PCa tumor spheroid outgrowth. In addition, lack of TRPM4 increased cell death in PCa tumor spheroids, a phenotype that is absent in two‐dimensional (2D) cancer cell systems. Lastly, absence of TRPM4 in PCa cells reduced extravasation and metastatic burden in a preclinical zebrafish cancer model. Taken together, our findings show that TRPM4 is an attractive therapeutic target in PCa and highlights the need for future development of pharmacological tools.

## Graphical abstract

Transient receptor potential melastatin‐4 (*TRPM4*) is overexpressed in prostate cancer (PCa). Knockout of *TRPM4* resulted in reduced PCa tumor spheroid size and decreased PCa tumor spheroid outgrowth. In addition, lack of TRPM4 increased cell death in PCa tumor spheroids. The absence of TRPM4 in PCa cells reduced extravasation and metastatic burden in a preclinical 3D zebrafish cancer model.

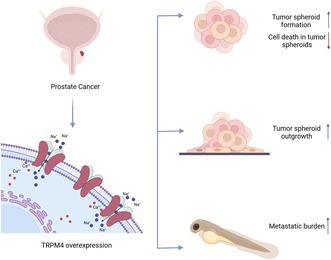

**Abbreviations**
2D: two‐dimensional
3D: three‐dimensional
BSA: bovine serum albumin
dpf: day postfertilization
EMT: epithelial‐to‐mesenchymal transition
KO: knockout
mRNA: messenger RNA
PCa: prostate cancer
Tg: thapsigargin
TRPM4: transient receptor potential melastatin‐4
ULA: ultra‐low attachment

## 1 Introduction

Prostate cancer (PCa) is the world's second most frequent cancer and the fifth leading cause of cancer death among men, with 1.5 million new cases and 397 000 estimated deaths per annum worldwide [1].

Severe disease progression is associated with lymphatic and distant metastatic spread. This poses major challenges to the clinical management of PCa due to a poor response to radical prostatectomy and radiation therapy upon metastasis [2]. For these reasons, an understanding of the dysregulated molecular key players and mechanisms that contribute to PCa progression, especially migration and invasion, is indispensable to develop therapeutic strategies targeting PCa.

Ion channels are attractive therapeutic targets, as they are often molecular switches that determine the fate of a cell, and they are pharmacologically accessible from outside the cell. In PCa, dysregulation of various ion channels contributes to cancer hallmarks, including reduced apoptosis, and increased proliferation, invasion, migration, epithelial‐to‐mesenchymal transition (EMT), and angiogenesis [3].

Mutations and dysregulation of the transient receptor potential melastatin‐4 (*TRPM4*) ion channel have been associated with immune and cardiac diseases as well as cancer [4, 5, 6, 7].

*TRPM4* mRNA is upregulated in several cancer entities but most prominently in PCa [8, 9]. *TRPM4* has been identified as a cancer driver gene in androgen‐independent PCa and has been associated with the risk of biochemical recurrence following radical prostatectomy [10, 11]. In addition, a high expression level of *TRPM4* is among the top risk factors in early PCa development [12]. TRPM4 plays a substantial role in PCa cellular malfunctions, including increased cell proliferation, migration, adhesion, EMT, cell cycle shift, and alterations of intracellular Ca^2+^ signaling [13, 14, 15, 16, 17, 18, 19, 20]. In addition, *TRPM4* is suppressed by proapoptotic protein p53 and dysfunctional p53 often associated with PCa results in increased TRPM4 activity [21, 22].

Intracellular Ca^2+^ signaling depends on the interplay of a plethora of Ca^2+^ transporting and binding enzymes. In PCa dysregulated Ca^2+^ signaling adds to cancer hallmarks, including increased proliferation and migration, invasion, and inability to induce cell death [23]. Upon activation by intracellular Ca^2+^, Na^+^ influx via TRPM4 decreases the plasma membrane potential and thereby the driving force for Ca^2+^ [24]. In prostate cancer cells, several TRPM4‐specific alterations of Ca^2+^ signaling have been reported [13, 15]. In addition, TRPM4 is part of the adhesome and thus affects cell migration [17, 25, 26, 27]. Other TRPM4‐related mechanisms may add to cellular malfunctions as TRPM4 has been reported to act via a multitude of interaction partners, and mechanisms including alterations of the β‐catenin pathway and localization in intracellular compartments [13, 19, 26, 27, 28, 29, 30, 31, 32, 33, 34].

Many TRPM4 blockers are known, but only a few are small molecule inhibitors that block TRPM4 in the submicromolar range [35, 36, 37, 38]. Halogenated anthranilic amides block TRPM4 currents with submicromolar potency and adequate selectivity [39, 40], and currently, these inhibitors are further developed [41, 42, 43]. However, in cellular assays, these inhibitors do not, or only to a minor extent, inhibit TRPM4‐specific effects on cellular malfunctions in PCa [18, 44].

Three‐dimensional tumor spheroids mimic aspects of solid tumors, including three‐dimensional tumor formation and tumor outgrowth, more realistically than 2D cell cultures [45]. In contrast to previous studies using two‐dimensional (2D) cell systems, we here investigate the role of TRPM4 in PCa progression using three‐dimensional (3D) PCa tumor spheroids and a preclinical *in vivo* zebrafish model [46] to access aspects of tumor formation and cancer development.

## 2 Materials and methods

### 2.1 Expression analyses

Publicly available mRNA microarray expression data of matched malignant and nonmalignant prostate tissue samples from 15 prostate cancer patients (GEO accession GSE69223, [47]) were used to analyze differentially expressed genes and expression of *TRPM4* in prostate cancer using GEO2R [48]. Log transformation was automatically detected by GEO2R, and *P*‐values were automatically adjusted according to Benjamini and Hochberg (false discovery rate).

### 2.2 Cell culture and *TRPM4* knockout clones

DU145 prostate cancer cells (CVCL_0105) from the American Type Culture Collection (ATCC, HTB‐81) were cultured in Minimum Essential Medium (Gibco, #32360026, Thermo Fisher Scientific, Reinach, Switzerland) supplemented with 10% (v/v) bovine calf serum (Sigma‐Aldrich, #8056, Buchs, Switzerland), 1% (v/v) L‐glutamine (200 mm, Gibco, #25030081), and 1% (v/v) MEM Non‐Essential Amino Acids Solution (100×, Gibco, #11140035). All cells were cultured at 37 °C in humidified air containing 5% CO_2_ and passaged every 3–4 days. DU145 *TRPM4* knockout (KO) clones had been generated as previously described, utilizing CRISPR/Cas9 and gRNAs targeting exons 2–4 of *TRPM4* [18].

Cells have been authenticated by morphology weekly and by STR profiling upon finalization of the experiments or 3 years after genetic modification and tested negatively for mycoplasma annually.

### 2.3 2D cell migration

To analyze migration potential, 50 000 DU145 cells were seeded into FluoroBlok Boyden chamber cell culture inserts (Corning, #351152, New York, NY, USA) using medium without serum. Migration inserts were then suspended in 24‐well tissue culture microplates (Corning, #353504) containing regular medium with 10% serum as chemoattractant and incubated for 72 h under normal culture conditions. Afterwards, cells were fixed in ice‐cold methanol and stained with DAPI (1 mg·mL^−1^, Sigma‐Aldrich, #MBD0015). Migrated cells were visualized by fluorescence light microscopy at excitation and emission wavelengths of 385/30 and 450/50 nm, respectively (4× objective, Echo Revolve, San Diego, CA, USA) and semiautomatically quantified (imagej, software version 1.53t) [49].

### 2.4 Spheroid culture

To form multicellular DU145 tumor spheroids, a total of 5000 cells per well were seeded into round‐bottomed ultra‐low attachment 96‐well microplates (faCellitate Biofloat, #F202003) as previously described [50]. Spheroids were grown under culture conditions described above, replacing half of the medium every 3–4 days. Spheroid formation and size were monitored by phase‐contrast light microscopy (10× objective, Echo Revolve) for at least 2 weeks. The development of spheroid size over time was quantified by measuring the area of the core spheroid at the largest diameter (echo pro, software version 6.4.2, Echo, San Diego, CA, USA).

### 2.5 Spheroid outgrowth

For spheroid outgrowth analysis, tumor spheroids were transferred to clear flat‐bottomed 24‐well tissue culture microplates (Greiner Bio‐One, #662160, Kremsmünster, Austria) by gentle micropipette aspiration, using cropped 1000 μL tips to reduce shear forces. Spheroids were transferred after stable formation on day 4 postseeding and cultured under the culture conditions described above. Tumor spheroid attachment and outgrowth were monitored by bright‐field light microscopy (4× objective, Echo Revolve) for at least 72 h. Spheroid outgrowth was quantified as the ratio of outgrowth area to core spheroid size (see above; echo pro, software version 6.4.2).

### 2.6 2D and spheroid cell death

Prior to analysis, tumor spheroid and conventional cell cultures (matched for seeding cell number) were entirely transferred to black flat‐bottomed 96‐well microplates (Thermo Scientific, #137101) by gentle micropipette aspiration using cropped 1000 μL tips, both including their respective culture media. Subsequent CellTox Green staining (Promega, #G8741, Dübendorf, Switzerland) was performed according to the manufacturer's instructions (endpoint method, concentrated dye reagent), and the resultant fluorescence intensities were measured at excitation and emission wavelengths of 485/20 and 535/25 nm, respectively (tecan spark microplate reader, software version 3.2, Tecan, Männedorf, Switzerland). Background and autofluorescence signals from microplates, media, and reagents were subtracted from all values. Fully lysed samples (lysis solution supplied) were used to determine maximum signal intensities and thus adjust for total cell number. Relative cell death was quantified as CellTox Green signal intensity per average maximum intensity of the corresponding group. For visualization of cell death, tumor spheroids were stained with calcein‐AM, propidium iodide and Hoechst 33342 (Sigma‐Aldrich, #CBA415) according to the manufacturer's instructions and imaged by fluorescence light microscopy at excitation and emission wavelengths of 470/40 and 525/50 nm, 530/40 and 590/40 nm, and 385/30 and 450/50 nm, respectively (10× objective, Echo Revolve).

### 2.7 Lentivirus production and transduction

Lentiviruses were produced using packaging plasmid PAX2, envelope plasmid pMD2G, and the insert pCDH‐EF1‐Luc2‐P2A‐tdTomato. The pCDH‐EF1‐Luc2‐P2A‐tdTomato was a gift from Kazuhiro Oka (Addgene plasmid #72486; http://n2t.net/addgene:72486; RRID:Addgene_72486, Addgene, Watertown, MA, USA). HEK‐293 T cells were transfected with the envelope, packaging, and insert plasmids using jetPRIME reagent (Polyplus, Illkirch‐Graffenstaden, France) according to the manufacturer's protocol. The final ratio of DNA to jetprime reagent was 1 : 2. Lentiviral supernatants were collected at 24, 48, and 72 h of transfection, pooled, and subsequently concentrated using PEG‐it virus precipitation solution (System Biosciences, Palo Alto, CA, USA) and according to the manufacturer's protocol. The lentiviral pellets were resuspended in PBS and stored in cryotubes at −80 °C. DU145 cells were transduced with the lentivirus using polybrene at 8 μg·mL^−1^ final concentration. Transduced cells were cultured for two passages and then sorted via flow cytometry for tdTomato‐positive cells.

### 2.8 Zebrafish maintenance, tumor cell implantation, and metastasis analysis

Experiments with zebrafish (*Danio rerio*) larvae were performed at the Institute of Anatomy of the University of Bern. The use of animals for husbandry was approved by the Animal Care and Experimentation Committee of the Canton of Bern, Switzerland (National License Number 35). The study was designed in accordance with ARRIVE guidelines and all experiments were performed in accordance with the guidelines and regulations approved by the Animal Care and Experimentation Committee of the Canton of Bern, Switzerland. Adult fish needed for breeding were maintained at the Institute of Anatomy according to previously described conditions [51]. The transgenic zebrafish line *Tg(fli1:eGFP)* was used for all extravasation experiments with DU145 and *TRPM4* KO cell lines. Freshly fertilized eggs were maintained at 28 °C in E3 medium and PTU was added at 1‐day postfertilization (dpf) to inhibit pigmentation. Thereafter, embryos were collected at 2 dpf and dechorionized with pronase (Roche, 2 mg·mL^−1^) for 5 min at 28 °C before injection. Cancer cells were collected with TripLE XP (ThermoFischer Scientific) for 5′, washed in FACS wash buffer [0.5% bovine serum albumin (BSA), 2 mm EDTA in PBS] and resuspended in injection medium (0.1% BSA, 0.5 mm EDTA and 2% polyvinylpyrrolidone in PBS). Cells were resuspended in injection medium at 200 000 cells·μL^−1^, manually loaded into borosilicate glass capillary needles (1 mm O.D: ×0.78 mm I.D.; #30‐0035, Harvard Apparatus, Holliston, MA, USA) and injected into the duct of Cuvier of 2 dpf zebrafish larvae. The dechorionized embryos were anesthetized using a 0.08 mg·mL^−1^ tricaine solution (Sigma) and placed on 1.5% agarose petri dishes and injected using a microinjector (Eppendorf, FemtoJet 4×, Hamburg, Germany). The injection parameters were 80–200 hPa pressure and 0.1–0.3 s time. The injected embryos were selected using a Nikon SMZ25 stereo microscope (Nikon Europe B.V., Amstelveen, The Netherlands) discarding uninjected and misinjected larvae and were maintained at 34 °C until endpoint (5 dpf). Larvae were anesthetized with tricaine, imaged using the NIS‐Elements Imaging software and euthanized by tricaine overdose and freezing method. Images were analyzed with Image J using a set of scripts publicly available on GitHub (https://github.com/Fredrigo87/ImageJ‐macros.git). Experiments were conducted in blinding conditions and at least 50 larvae were injected per condition.

### 2.9 Ca^2+^ imaging

Cells were grown on cover slips and loaded with FURA‐2AM (Thermo Fisher, F1221, Reinach, Switzerland) for 15 min at 37 °C and 5% CO_2_. Coverslips were transferred into custom‐built measurement chambers. After the initial wash with 0.5 mm Ca^2+^‐Ringer solution, cells were allowed to equilibrate for 5 min before the measurement started. During the measurement, solutions were changed according to the application scheme in the relevant figures. Imaging solutions contained: 155 mm NaCl, 4.5 mm KCl, 2 mm MgCl_2_, 10 mm D‐glucose, and 5 mm HEPES. Ca^2+^ was adjusted as indicated. 0 mm Ca^2+^ was adjusted by the addition of 1 mm EGTA. To ensure passive store depletion, 1 μm thapsigargin (Tg, Thermo Fisher, T7459) was used as indicated. This method was used earlier [21].

### 2.10 Statistical analyses

All statistical analyses were performed using graphpad prism version 10.2.3 (Boston, MA, USA). Experimental data were tested for homogeneity of variances using an *F*‐test and for normal distribution using the D'Agostino and Pearson tests. Differences between two nonindependent samples were analyzed using a two‐tailed paired *t*‐test. Differences between multiple independent samples were analyzed using ordinary one‐way analysis of variance (one‐way ANOVA) followed by Dunnett's *post‐hoc* test. The significance level (α) was set at a value of 0.05. All cell culture experiments were repeated at least four times in triplicate.

## 3 Results and Discussion

### 3.1 *TRPM4* gene expression levels in prostate cancer tissue samples and TRPM4 in 2D migration and cell death assays

**Fig. 1:**
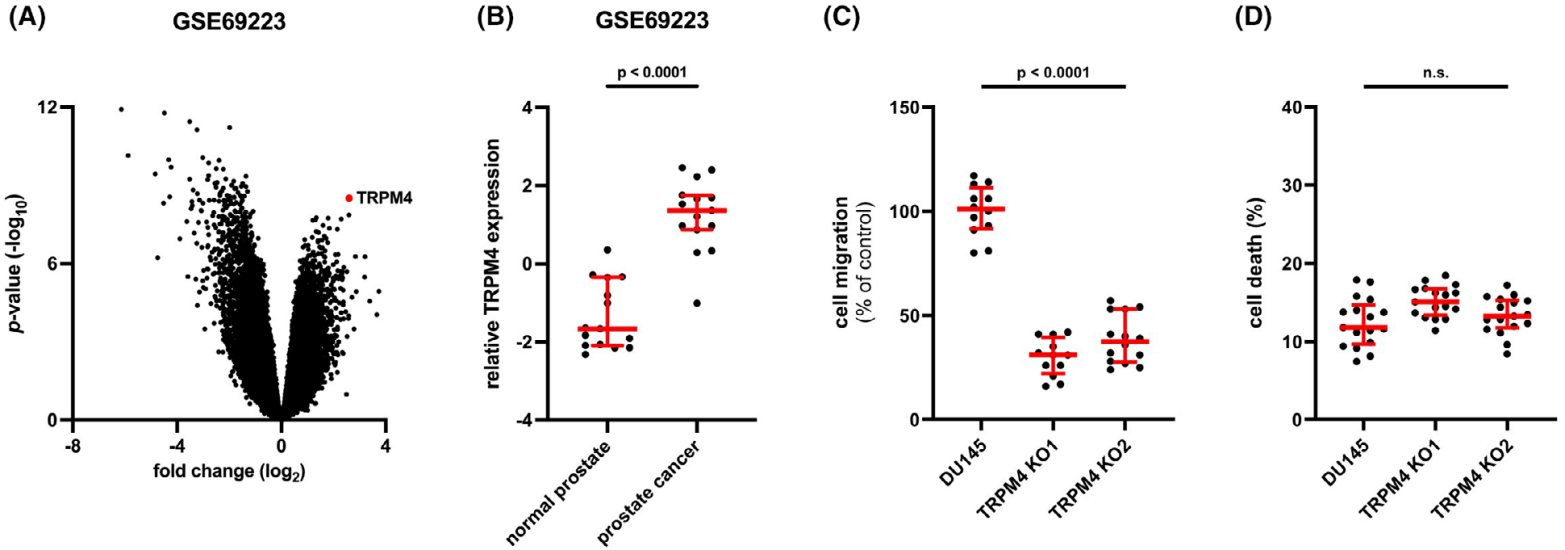
Expression of transient receptor potential melastatin‐4 (*TRPM4*) in prostate cancer (PCa) patient samples and migration and cell death in 2D cell assays. (A) Volcano plot of gene expression in matched malignant and nonmalignant prostate tissue samples from 15 PCa patients (GSE69223, mRNA microarray). (B) Relative expression of *TRPM4* from data in (A). Differences between samples were analyzed using a two‐tailed paired *t*‐test. (C) Statistical analysis of cell migration of DU145 cells and corresponding *TRPM4* knockout (KO) clones in FluoroBlok Boyden chambers 72 h postseeding (*n* = 4). (D) Cell death was quantified as the relative intensity of CellTox Green staining in DU145 cells and corresponding *TRPM4* KO clones on day 3 postseeding (*n* = 4). All individual values are plotted as dots in the graph. The median is shown as a red line, whiskers represent the interquartile range. Significance levels for data from both clones in C and D are given relative to DU145 cells. Differences between samples were analyzed using one‐way ANOVA followed by Dunnett's *post‐hoc* test.

Meller et al. [47] performed an mRNA microarray of ~ 6350 genes in 15 matching nonmalignant and malignant prostate tissue samples from PCa patients with a T2 or T3 tumor staging. The volcano plot from a GEO2R analysis demonstrates that *TRPM4* mRNA expression is more than sixfold increased in malignant tissues and is among the top significantly upregulated genes (*P*‐value ~ 3 × 10^−9^) (Fig. 1A,B).

CRISPR/Cas mediated knockout of *TRPM4* in PCa cell line DU145 significantly reduced migration in a single cell Boyden chamber migration assay in two *TRPM4* knockout clones (*TRPM4* KO1 and *TRPM4* KO2) (Fig. 1C). This is in line with previous reports [14, 15, 16, 17]. Cell death was analyzed and quantified as the relative intensity of CellTox Green staining in DU145 cells and corresponding *TRPM4* KO clones. Absence of TRPM4 did not alter cell death in a 2D cell death assay (Fig. 1D). So far, no role for TRPM4 in the cell death of PCa cells has been reported, although there is evidence from other systems that TRPM4 can be involved in cell death [33, 52, 53, 54].

### 3.2 *TRPM4* knockout affects tumor spheroid formation, size, and adhesion

**Fig. 2:**
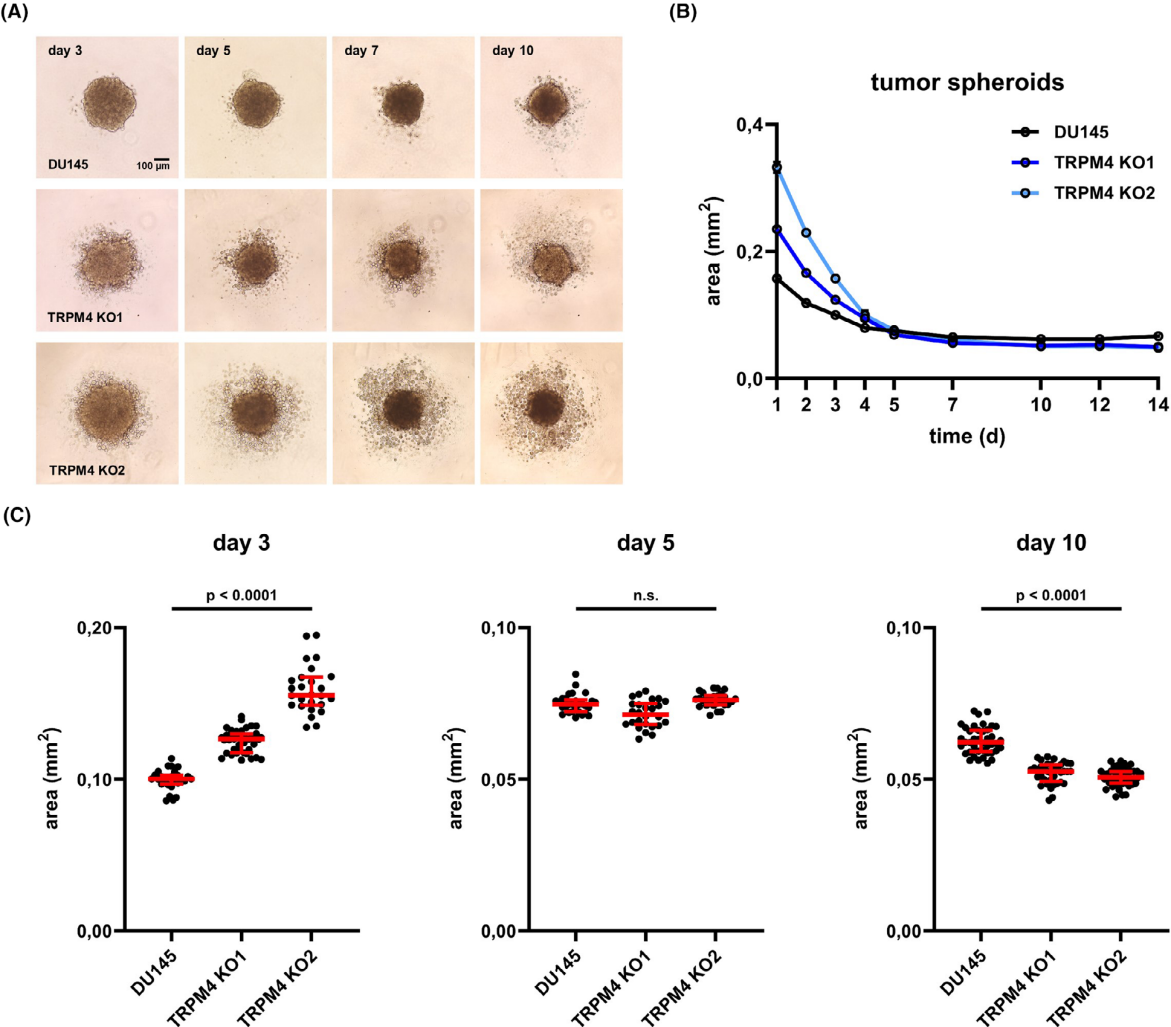
Transient receptor potential melastatin‐4 (*TRPM4*) knockout (KO) affects tumor spheroid formation, size and adhesion. (A) Representative microscopic images of tumor spheroids from DU145 cells and corresponding *TRPM4* KO clones (*n* = 4). Scale bar indicates 100 μm. (B) Development of spheroid size over time was quantified by measuring the area of the core spheroid at the largest diameter (*n* = 4). (C) Statistical analysis of spheroid size on days 3, 5, and 10 postseeding. All individual values are plotted as dots in the graph. The median is shown as a red line, whiskers represent the interquartile range. Significance levels for data from both clones in C are given relative to DU145 cells. Differences between samples were analyzed using one‐way ANOVA followed by Dunnett's *post‐hoc* test.

To generate multicellular tumor spheroids, DU145 prostate cancer cells and corresponding *TRPM4* KO1 and *TRPM4* KO2 were dissociated, counted, and seeded into ultra‐low attachment (ULA) microplates. Solid three‐dimensional tumor spheroids were formed within 3–4 days and remained stable for several weeks (Fig. 2A,B). Notably, tumor spheroids from both *TRPM4* KO clones contained loose or shed cells around the spheroid core (Fig. 2A). Knockout of *TRPM4* affected tumor spheroid formation and the development of spheroid size over time. During initial spheroid formation on days 1–3, *TRPM4* KO clones showed an apparently less dense spheroid core that was increased in size pointing towards reduced tumor spheroid formation (Fig. 2C, left panel). Both, shed cells and larger, less dense spheroid cores in KO conditions may reflect the decreased cell adhesion when TRPM4 is absent that has been shown in image and electrical impedance based 2D assays [18].

No significant differences in spheroid size were observed by day 5 (Fig. 2C, middle panel). At later time points from day 10, *TRPM4* KO clones showed a significantly decreased spheroid core size (Fig. 2C, right panel), probably caused by reduced adhesion and proliferation that has been shown earlier in 2D systems when TRPM4 is absent or reduced [13, 14, 15, 16, 17, 18].

### 3.3 *TRPM4* knockout reduces tumor spheroid outgrowth

**Fig. 3:**
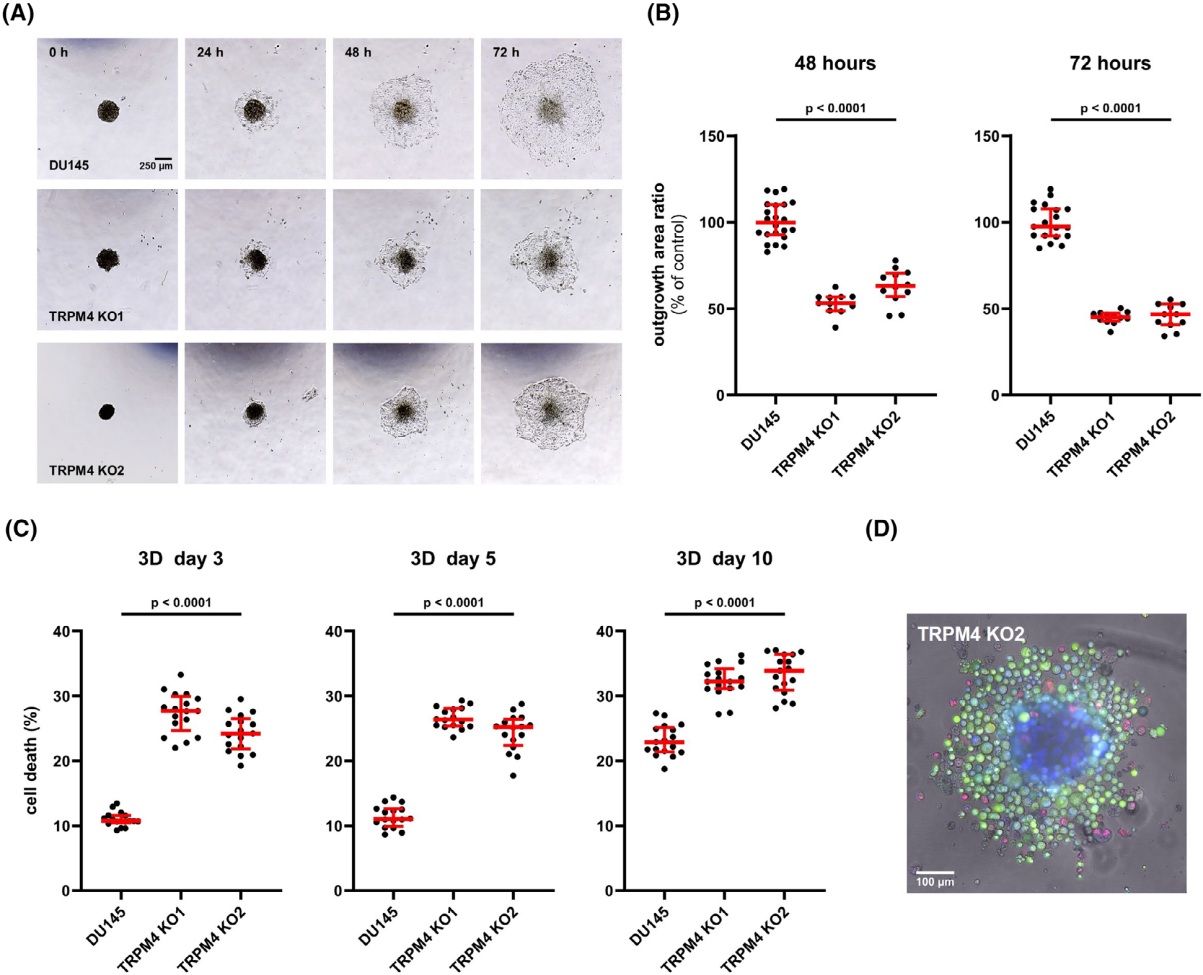
Transient receptor potential melastatin‐4 (*TRPM4*) knockout (KO) reduces tumor spheroid outgrowth and increases cell death in tumor spheroids. (A) Representative microscopic images of tumor spheroid outgrowth from DU145 cells and corresponding *TRPM4* KO clones (*n* = 4). Scale bar indicates 250 μm. (B) Spheroid outgrowth was quantified as the ratio of outgrowth area to core spheroid size at 48 and 72 h. All individual values are plotted as dots in the graph. The median is shown as a red line, whiskers represent the interquartile range. (C) Cell death was quantified as the relative intensity of CellTox Green staining in tumor spheroids from DU145 cells and corresponding *TRPM4* KO clones on days 3, 5, and 10 postseeding (*n* = 4). All individual values are plotted as dots in the graph. The median is shown as a red line, whiskers represent the interquartile range. Significance levels for data from both clones in B and C are given relative to DU145 cells. Differences between samples were analyzed using one‐way ANOVA followed by Dunnett's *post‐hoc* test. (D) Representative fluorescence microscopic image of a tumor spheroid from DU145 *TRPM4* KO2 cells on day 5 postseeding (*n* = 4). Live cells were stained with calcein‐AM (green), dead cells with propidium iodide (red), and the nuclei of all cells with Hoechst 33342 (blue). Scale bar indicates 100 μm.

To further characterize the contribution of TRPM4 to cell adhesion and migration, tumor spheroid outgrowth was analyzed. After stable formation on day four postseeding, spheroids were transferred to flat‐bottomed tissue culture microplates, with cropped micropipette tips to reduce shear forces. Notably, upon transfer already at day 4 tumor spheroids from *TRPM4* KO clones appear smaller than those from parental cells, probably due to decreased proliferation, adhesion, and the loss of shed cells. Upon attachment, spheroid outgrowth was quantified as the ratio of outgrowth area to core spheroid size to account for potential size differences. Tumor spheroid outgrowth was observed for parental DU145 cells and corresponding *TRPM4* KO clones within 24 h and continued for a total of at least 72 h (Fig. 3A). *TRPM4* knockout significantly reduced spheroid outgrowth at 48 and 72 h (Fig. 3B), demonstrating its previously described potential role in cell adhesion and migration in 3D tumor spheroid outgrowth.

### 3.4 *TRPM4* knockout increases cell death in tumor spheroids

**Fig. 4:**
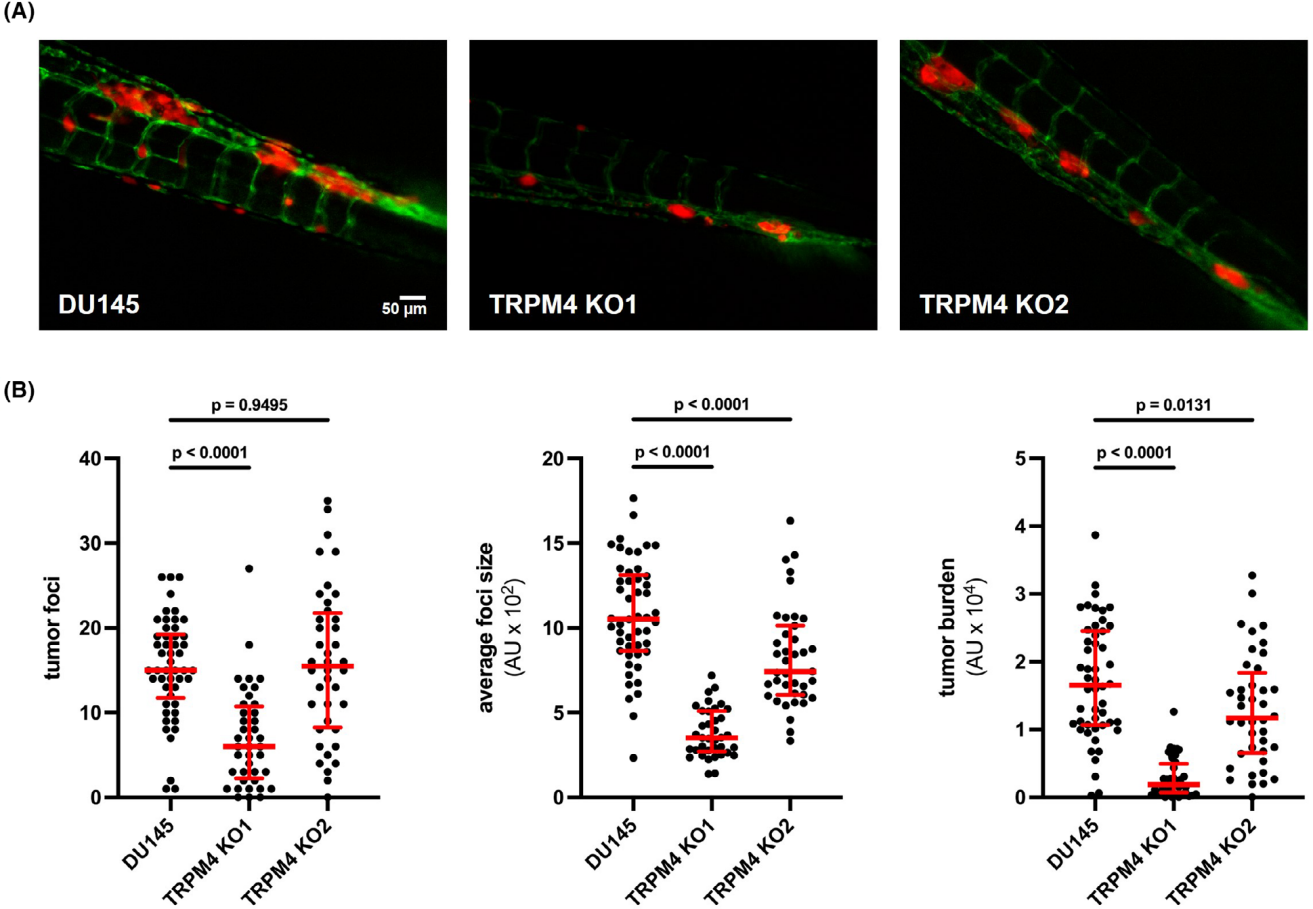
Transient receptor potential melastatin‐4 (*TRPM4*) knockout (KO) reduces extravasation and metastatic burden of PCa cells in zebrafish xenograft model. (A) Representative stereomicroscopic fluorescence images of metastatic colonization of 2 day postfertilization (dpf) zebrafish larvae with endothelial reporter *tg*(*Fli:GFP*), after injection of DU145 cells (*n* = 50) and corresponding *TRPM4* KO clones (both *n* = 40) stably expressing tdTomato. Scale bar indicates 50 μm. (B) Tumor foci (left), average foci size (middle) and overall tumor burden (right) were measured at day 3 postinjection. Foci number and size were determined by automatic segmentation of the tdTomato fluorescence intensity within the tail of the injected zebrafish larvae. Tumor burden was quantified as average tdTomato fluorescence intensity within the same region. Fifty larvae injected with DU145 cells and 40 larvae injected with each of the corresponding *TRPM4* KO clones were analyzed. All individual values are plotted as dots in the graph. The median is shown as a red line, whiskers represent the interquartile range. Significance levels for data from both clones in B are given relative to DU145 cells. Differences between samples were analyzed using one‐way ANOVA followed by Dunnett's *post‐hoc* test.

TRPM4 contributes to several cancer hallmarks in PCa including increased proliferation, migration, and EMT transition; however, no findings on a putative role of TRPM4 in cell death of PCa cells have been published. In addition, we did not find a change in cell death in a 2D cell death assay when TRPM4 was absent (Fig. 1D). In contrast, tumor spheroids from both *TRPM4* KO clones showed sustained and significantly increased cell death from day 3 to 10 postseeding when compared to tumor spheroids from parental DU145 cells (Fig. 3C). Increased cell death could add to reduced tumor spheroid size when TRPM4 is absent. Figure 3D shows an example of live/dead cell staining demonstrating that most cells around the spheroid core of *TRPM4* KO clones are apparently alive (Fig. 4B).

### 3.5 *TRPM4* knockout reduces extravasation and metastatic burden in zebrafish xenografts

To investigate the metastatic and extravasation potential *in vivo*, DU145 cells and corresponding *TRPM4* KO clones were injected into a zebrafish xenograft model. This model enables effective evaluation of invasion and extravasation of human PCa cells and interaction with the vasculature at the single cell level effectively utilizing a combination of fluorescent vasculature and transparent zebrafish embryos [55]. Perivascular tumor cell extravasation was detected in multiple foci around the dorsal aorta, caudal vein as well as intersegmental vessels (Fig. 4A). We observed that the number of tumor foci was reduced in *TRPM4* KO cells (*P* < 0.0001 and *P* > 0.9 for KO1 and KO2, respectively, Fig. 4B). Interestingly, despite a similar amount of tumor foci in KO2, the average size of tumor foci was significantly smaller, as confirmed by a significantly reduced overall tumor burden in both conditions (*P* < 0.0001 and *P* = 0.0131 for KO1 and KO2, respectively). Overall, DU145 *TRPM4* KO cells showed a reduced capacity to extravasate and form metastases *in vivo*.

The *in vivo* data show that TRPM4 is required for proficient metastatic colonization of an advanced prostate cancer cell model. This data is in line with the presented *in vitro* data, showing a reduced local outgrowth and increased cell death of DU145 *TRPM4* KO cells in 3D cancer spheroids.

Interestingly, while both tested *TRPM4* KO clones resulted in a significantly reduced metastatic burden, KO1 had a more dramatic effect, with few and smaller cancer foci in the fish tail. KO2 instead showed a number of extravasation events comparable to control cells but resulting in significantly smaller metastases. Ca^2+^ imaging experiments demonstrate that in *TRPM4* KO1 rate and peak of store‐operated Ca^2+^ entry is elevated compared to DU145 (Fig. S1), coherent with earlier findings from experiments with siRNA [15]. In *TRPM4* KO2, Ca^2+^ signaling is decreased probably due to compensatory mechanisms. This data suggests that a TRPM4 mediated decrease of Ca^2+^ signaling may add to the metastatic burden, yet other mechanisms add to the TRPM4 related pathophysiology.

Overall, our findings highlight TRPM4 as a key driver in early metastatic events, which include increased survival in circulation, effective extravasation, and establishment of local metastatic foci.

## 4 Conclusion

*TRPM4* is among the top significantly upregulated genes in PCa and the absence of *TRPM4* reduced several cancer‐related functions in PCa cells in 2D PCa systems. Here, we show that in a 3D PCa tumor spheroid model system, knockout of *TRPM4* in PCa cells reduced tumor spheroid size, and outgrowth and increased cell death. In a zebrafish cancer model, *TRPM4* knockout reduced metastatic burden and extravasation. Future inhibitors will have to prove that they block TRPM4‐related effects in 3D cancer systems.

Taken together, our findings indicate that TRPM4 is an attractive putative therapeutic target in PCa. Further research is needed to understand the involved mechanisms and to manage the pharmaceutical approach in 3D cancer systems.

## Conflict of interest

The author(s) declare financial support was received for the research, authorship, and/or publication of this article. The authors declare that the research was conducted in the absence of any commercial or financial relationships that could be construed as a potential conflict of interest.

## Author contributions

FB, SS, FLM, SJ, IP, MK, and CP made substantial contributions to the conception or design of the work; FB, SS, FLM, SJ, and IP made substantial contributions to the acquisition and analysis, and FB, SS, FLM, SJ, IP, MK, and CP to the interpretation of data for the work. FB, SS, FLM, and CP drafted the work and FB, SS, FLM, IP, MK, and CP reviewed it critically for important intellectual content. FB, SS, FLM, SJ, IP, MK, and CP finally approved the version to be published and are accountable for all aspects of the work in ensuring that questions related to the accuracy or integrity of any part of the work are appropriately investigated and resolved.

## Supporting information


**Fig. S1.** Fura‐2 AM‐based Ca^2+^ imaging of DU145 and transient receptor potential melastatin‐4 (*TRPM4*) knockout (KO) cells.

## Data Availability

The original contributions presented in the study are included in the article/Supplementary Material or are available in the online data depository (https://doi.org/10.5281/zenodo.14534116).
