# Supplementary material for: TRPM4 contributes to cell death in prostate cancer tumor spheroids, and to extravasation and metastasis in a zebrafish xenograft model system
Source: Mol Oncol. 2025 Jan 16;19(5):1299–309. doi: 10.1002/1878-0261.13795 (PMC12077273; doi:10.1002/1878-0261.13795)
Supplement: Supplementary file 1 — Fig. S1. Fura‐2 AM‐based Ca2+ imaging of DU145 and transient receptor potential melastatin‐4 (TRPM4) knockout (KO) cells. [file MOL2-19-1299-s001.zip › Figure Legends.docx]

**Figure Legends of Supporting Information**

**Supplementary Figure 1: Fura-2AM-based Ca^2+^ imaging of DU145 and transient receptor potential melastatin-4 (TRPM4) knockout (KO) cells.**

(A) Fluorescence ratio development over time in DU145 (n = 88) and TRPM4 KO1 (n = 94) and TRPM4 KO2 (n = 128) in a standard Ca^2+^ re-addition protocol (solution changes indicated on top of the graph). (B) Quantification of Ca^2+^ influx rate, Ca^2+^ peak, and Ca^2+^ plateau from (A). Data are plotted as mean±SEM, analyzed with a one-way ANOVA and significance is indicated by asterisks *p < 0.05, **p < 0.01, ***p < 0.001 and ****p < 0.0001.
