# Supplementary figures and images for: TRPM4 contributes to cell death in prostate cancer tumor spheroids, and to extravasation and metastasis in a zebrafish xenograft model system
Source: Mol Oncol. 2025 Jan 16;19(5):1299–309. doi: 10.1002/1878-0261.13795 (PMC12077273; doi:10.1002/1878-0261.13795)

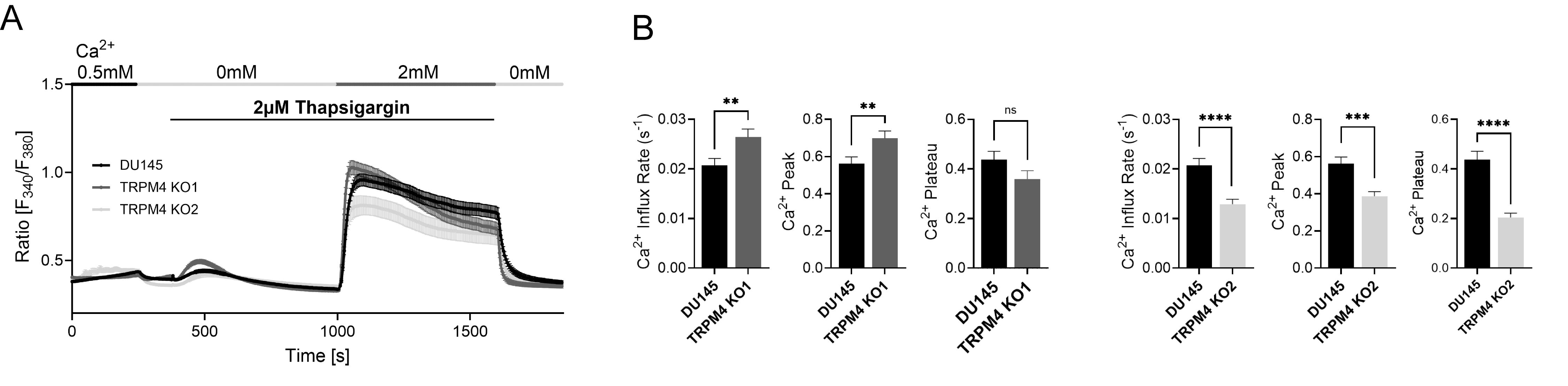

Supplement: Supplementary file 1 — Fig. S1. Fura‐2 AM‐based Ca2+ imaging of DU145 and transient receptor potential melastatin‐4 (TRPM4) knockout (KO) cells. [file MOL2-19-1299-s001.zip › mol213795-sup-0001-FigureS1.tif]
